# Cerebral Dopamine Neurotrophic Factor Diffuses Around the Brainstem and Does Not Undergo Anterograde Transport After Injection to the Substantia Nigra

**DOI:** 10.3389/fnins.2019.00590

**Published:** 2019-06-11

**Authors:** Katrina Albert, Juho-Matti Renko, Kert Mätlik, Mikko Airavaara, Merja H. Voutilainen

**Affiliations:** ^1^Institute of Biotechnology, HiLIFE, University of Helsinki, Helsinki, Finland; ^2^Division of Pharmacology and Pharmacotherapy, Faculty of Pharmacy, University of Helsinki, Helsinki, Finland; ^3^Neuroscience Center, HiLIFE, University of Helsinki, Helsinki, Finland

**Keywords:** cerebral dopamine neurotrophic factor, substantia nigra, Parkinson’s disease, striatum, diffusion, transport

## Abstract

Cerebral dopamine neurotrophic factor (CDNF) has shown therapeutic potential in rodent and non-human primate models of Parkinson’s disease by protecting the dopamine neurons from degeneration and even restoring their phenotype and function. Previously, neurorestorative efficacy of CDNF in the 6-hydroxydopamine (6-OHDA) model of Parkinson’s disease as well as diffusion of the protein in the striatum (STR) has been demonstrated and studied. Here, experiments were performed to characterize the diffusion and transport of supra-nigral CDNF in non-lesioned rats. We injected recombinant human CDNF to the substantia nigra (SN) of naïve male Wistar rats and analyzed the brains 2, 6, and 24 h after injections. We performed immunohistochemical stainings using an antibody specific to human CDNF and radioactivity measurements after injecting iodinated CDNF. Unlike the previously reported striatonigral retrograde transport seen after striatal injection, active anterograde transport of CDNF to the STR could not be detected after nigral injection. There was, however, clear diffusion of CDNF to the brain areas surrounding the SN, and CDNF colocalized with tyrosine hydroxylase (TH)-positive neurons. Overall, our results provide insight on how CDNF injected to the SN may act in this region of the brain.

## Introduction

Cerebral dopamine neurotrophic factor (CDNF) is a member of the evolutionarily conserved group of proteins with its homolog mesencephalic astrocyte-derived neurotrophic factor (MANF) (Lindholm et al., 2007; Lindholm and Saarma, 2010). In line with expression data from rodents, CDNF transcript is present in human brain as well as peripheral tissues (Lindholm et al., 2007). Despite intracellular localization of endogenous CDNF, *in vivo* data with intracerebral injections of recombinant human CDNF shows that it can protect dopamine neurons, and more importantly, restore the functionality of the nigrostriatal dopamine system after toxin-induced degeneration in rodent models of Parkinson’s disease (PD) (Lindholm et al., 2007; Voutilainen et al., 2011; Airavaara et al., 2012). There is also evidence that CDNF may be involved in endoplasmic reticulum (ER) stress (Voutilainen et al., 2017). CDNF is a protein recently used in PD research and tested in phase I-II clinical trials; it has a structure and a putative mode of action that is distinct from glial cell-derived neurotrophic factor (GDNF) and neurturin, as well as other neurotrophic factors. Specifically, MANF, and also CDNF, have a saposin-like N-terminal domain which is thought to interact with lipids, and indeed MANF has been shown to interact with sulfatides (Bai et al., 2018). The C-terminus of MANF has an α-helical structure (Parkash et al., 2009) with a cysteine bridge that is similar to other proteins that function at the ER (Ellgaard and Ruddock, 2005). During ER stress, the level of MANF is increased and it is able to bypass the ER-stress induced translational block (Apostolou et al., 2008), and the same may be happening for CDNF, though this needs further studies. In general, extracellularly applied protein and endogenously expressed MANF are likely acting differently (Matlik et al., 2015; Hao et al., 2017), and in relation to this study, exogenously administered recombinant CDNF can have a different mechanism and effects than in its endogenous location.

Radiolabelled CDNF has been shown to be transported to the substantia nigra (SN) pars compacta within 24 h after an injection to the striatum (STR) of non-lesioned rats (Voutilainen et al., 2011). A recent study described the spread and retrograde transport of CDNF after striatal injection, showing that CDNF could be detected inside tyrosine hydroxylase (TH)+ neurons of the SN in non-lesioned rats, but not in 6-hydroxydopamine (6-OHDA)-lesioned rats where the striatal endings of TH+ neuron fibers were damaged (Matlik et al., 2017). Inside the striatum, recombinant human CDNF was observed to reside both extracellularly and in the cytoplasm of striatal neurons (Matlik et al., 2017). However, it has not been studied whether CDNF is transported or taken up by specific types of neurons after nigral injection.

## Materials and Methods

### Animals

Young adult male Wistar rats (200–250 g) were used in the experiments. Rats were housed in groups of four in a 12 h light/dark cycle, with *ad libitum* access to food and water. All surgeries and behavioral assays were carried out at the University of Helsinki Laboratory Animal Centre. All animal experiments were approved by the Finnish National Board of Animal Experiments (ESAVI/5459/04.10.03/2011 and ESAVI/7812/04.10.07/2015) and were carried out according to the European Community guidelines for the use of experimental animals.

### Stereotaxic Surgeries

For CDNF injections, rats were anesthetized with isoflurane (4% induction, 2.5–3% maintenance) and placed into the stereotaxic frame (Stoelting). After disinfecting the skin, >0.1 ml of lidocaine with adrenaline (10 mg/ml, Orion Pharma) was injected under the scalp. A cut was made along the top of the head to expose the skull and burr holes were made with a high-speed drill. A 10 μl Hamilton syringe with a 26G steel needle attached was used to inject recombinant human CDNF (Biovian Oy, Finland) into the SN (A/P -5.4, M/L +2.0, D/V -7.2) at a concentration of 0.75 μg/μl. The volume for each injection was 4 μl with a flow rate of 0.5 μl/min and the needle was left to sit for 5 min after the injection. Total amount of protein was 3 μg to the SN. For the diffusion studies in the naïve rat brain, CDNF was injected to the SN in the same coordinates as above at a volume of 1 or 4 μl and a flow rate of 0.5 μl/min. The amount of protein in each injection was 3 μg regardless of volume. After stitching the wound, carprofen (5 mg/kg s.c., Pfizer) was given for post-operative pain. Rats were placed in a recovery box and then returned to their home cage upon awakening.

### Preparation, Injection, and Analysis of ^125^I-CDNF

Cerebral dopamine neurotrophic factor (1 μg, Biovian) was dissolved in 30 μl of 250 mM phosphate buffer, pH 7.5, and 2.7 μl of carrier-free ^125^I-Na (5 mCi/14 μl, Perkin Elmer) was added. First, 5 μl of lactoperoxidase (50 μg/ml, Sigma) was added to start the reaction. Next, 5 μl of H_2_O_2_ was added two times at 10 min intervals and the reaction mixture was incubated for 20 min at room temperature. The reaction was stopped with 150 μl of stop solution (0.1 M phosphate buffer, pH 7.5, containing 0.1 M NaI, 0.42 M NaCl) and 25 μl of 2.5% BSA in PBS. The ^125^I-labeled CDNF was separated from free iodine by gel filtration using Sephadex G-25 columns (PM10; GE Healthcare).

The specific activity for ^125^I-CDNF was 10^8^ cpm/ug of CDNF protein and CDNF concentration was 0.5 μg/ml. A bolus of 0.5^∗^10^6^ cpm of CDNF labeled with ^125^I was injected (5 ng of protein in total) as described above, using a 10 μl Hamilton syringe and a flow rate of 0.5 μl/min into the STR (A/P +1.0, M/L +2.7 D/V -5.0) or SN (A/P -5.4, M/L +2.0, D/V -7.2) of adult male Wistar rats. The rats were transcardially perfused 24 h later with PBS to remove the blood. The brain was removed and dissected immediately after the perfusion. The radioactivity of the samples was measured on the Wizard 3 1480 Automatic Gamma Counter (Perkin Elmer, Wallac). Values are expressed in cpm/mg of wet tissue. For the experiment with unlabelled CDNF, the injections to the SN were performed as described above and an additional 10 μg (2000 fold molar excess) or 50 μg (10 000 fold molar excess) of the unlabelled CDNF protein was injected to the same location. To calculate the percentage of total radioactivity, the amount of radioactivity in each dissected brain area was divided by the summed radioactivity in all brain areas (total radioactivity). 5–6 rats were used for each experiment.

### Tissue Collection and Processing

Two to twenty-four hours after CDNF injections, rats were deeply anesthetized with a lethal dose of pentobarbital (90 mg/kg, i.p., Orion Pharma). They were perfused transcardially with PBS, and then with 4% paraformaldehyde (PFA). The brains were removed and placed in 4% PFA overnight, and then transferred to a 20% sucrose solution and stored in +4°C. The brains were frozen in a cryostat (Leica CM3050) and 40 μm-thick coronal sections were collected from the whole brain. The sections were collected into anti-freeze buffer (40% 0.5 M PBS, 30% ethyleneglycol, 30% glycerol) into sets of six in a 24-well plate.

For the diffusion study, naïve rats injected with CDNF to the SN were euthanized 2, 6, or 24 h after the injection using the same perfusion procedure as described above (*N* = 2/time point). The brains were removed and embedded in paraffin and cut in 5 μm sagittal sections from 1.40 to 3.40 mm relative to bregma, taking every 10th section. The sections were put onto slides and stored in +4°C.

### Immunohistochemistry and Immunofluorescence

For chromagen staining of CDNF, sections were removed from anti-freeze buffer, rinsed with TBS and then heated in 10 mM citrate buffer, pH 6, with 0.05% Tween 20 at +80°C for 30 min. Sections were left to cool at room temperature for 15 min then rinsed with TBS-T (0.1% Tween 20) and blocked with goat serum (S1000, Vector) in TBS-T (0.015% serum in TBS-T) for 20 min. The sections were then incubated in the primary antibody solution (rabbit-anti-human CDNF, 1:500, stock solution 0.4 mg/mL, Icosagen) overnight at +4°C. The next day the sections were incubated in the secondary antibody solution (goat-anti-rabbit, 1:200, BA1000 Vector) for 1 h and then transferred into the avidin-biotin complex solution (ABC kit, Vector) for 30 min and lastly reacted with DAB. The sections were then rinsed with TBS, put onto glass microscope slides, dried overnight, dehydrated, mounted and coverslipped with Coverquick 2000 (Q PATH). Five rats per group were used.

For paraffin sections, after deparaffinization, sections underwent antigen retrieval by heating them up in citraconic anhydride (0.05% citraconic anhydride in MilliQ^®^ water, pH 7.4) without boiling. Slides were then cooled and endogenous peroxidase activity was quenched using 3% H_2_O_2_ in TBS for 30 min at room temperature. Slides were washed with TBS then sections were encircled with a PAP pen, washed with TBS-T and blocked with 1.5% normal horse serum (Vector) in TBS-T for approximately 20 min. The primary antibody (rabbit-anti-human CDNF, 1:1000, stock solution 0.4 mg/mL, Icosagen) was then added onto the sections and the slides were incubated at +4°C overnight. The next day, the sections were incubated in the secondary antibody solution (horse-anti-rabbit 1:200, Vector) for 1 hr at room temperature and then in the avidin-biotin complex solution (ABC kit, Vector) for 30 min. Slides were rinsed with TBS-T and then the sections were incubated with DAB for 5 min, rinsed with TBS, dehydrated, mounted, and coverslipped with Coverquick 2000 (Q PATH).

For fluorescent double staining, the sections underwent deparaffinization, antigen retrieval and blocking as described above (except blocking solution used goat serum). The solution containing the primary antibodies (rabbit-anti-human CDNF, 1:1000, stock solution 0.4 mg/mL, Icosagen, and mouse-anti-TH, 1:500, Chemicon, MAB318 or mouse-anti-parvalbumin, 1:1000, Merck, MAB1572) was placed on the sections after which they were incubated at +4°C overnight. The next day, the sections were incubated in blocking solution containing secondary antibodies (goat-anti-rabbit AlexaFluor488, A-11034, ThermoFischer Scientific, and goat-anti-mouse AlexaFluor568, A-11004, ThermoFischer Scientific, 1:200) for 2 h at room temperature. Slides were then washed with TBS-T, briefly with MilliQ^®^ water, coverslipped with Vectashield HardSet mounting medium with DAPI (Vector, H-1500) and after drying briefly at room temperature stored in +4°C.

For the confocal images, the fluorescent images stacks were acquired using the TCS SP5 Confocal Microscope equipped with LAS AF 1.82 (Leica Microsystems). The objective was Leica HCX PL APO 63_/1.3 GLYC CORR CS (21°C). The lasers used were DPSS 561 nm/20 mW, OPSL 488 nm/270 mW, and diode 405 nm/50 mW, with the beam splitter QD 405/488/561/635. The images were analyzed with the Leica LAS AF software. A total of 6 rats were used to take representative images from. The number of puncta inside the cells was analyzed qualitatively from 10 images taken/rat. This analysis was performed by counting the number of cells per image that were positive for the CDNF puncta.

### Statistical Analysis

Graphs were made in GraphPad Prism 6 (GraphPad Software Inc.) and statistical analyses were performed in SPSS 22 (IBM). All results are expressed as the mean ± SEM and considered to be significant at *p* < 0.05. *P*-values are reported from statistical tests used.

## Results

### Diffusion of ^125^I-CDNF After STR or SN Administration in Naïve Rats

**FIGURE 1:**
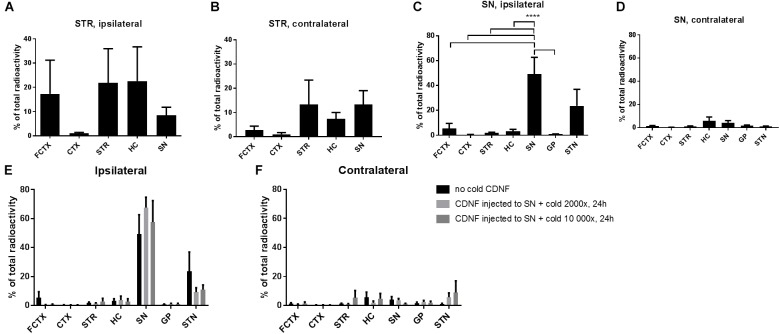
Diffusion of ^125^I CDNF when injected to the STR or SN of naive rats. **(A,B)** Percentage of the total radioactivity (calculated based on radioactivity of all brain areas measured) after injecting radiolabeled CDNF (^125^I-CDNF) into the STR of naive rats and measuring 24 h later, ipsilateral regions **(A)** and contralateral **(B)**. **(C,D)** Percentage of the total radioactivity (calculated based on radioactivity of all brain areas measured) after injecting radiolabeled CDNF into the SN of naive rats and measuring 24 h later, ipsilateral regions **(C)** and contralateral **(D)**. **(E,F)** 2000× or 10 000× molar excess of cold (unlabeled) CDNF was given together with radiolabeled CDNF to SN of naive rats and measured 24h later. Ipsilateral regions **(E)** and contralateral **(F)**. (*n* = 5–6/brain region) Bars represent mean ± SEM. ^∗∗∗∗^*p* = < 0.0001. FCTX, frontal cortex; CTX, cortex; STR, striatum; HC, hippocampus; SN, substantia nigra; GP, globus pallidus; STN, subthalamic nucleus.

To quantitatively study the diffusion and transport of intrastriatally injected CDNF to other brain areas, and to repeat our previously published data (Voutilainen et al., 2011), as well as have a positive control, ^125^I-labeled CDNF was injected to the STR of rats, and 24 h later dissected brain areas were measured in a gamma counter (Figure 1A,B). CDNF spread intensely to the frontal cortex of the injected side, while there was little spread of ^125^I-CDNF to other cortical areas. CDNF also spread well to the ipsilateral and contralateral hippocampus, and SN (Figure 1A,B).

Next, we wanted to analyze CDNF diffusion after nigral administration and possible anterograde transport from SN to STR which has not been studied before. CDNF labeled with ^125^I was injected into the SN of rats and brain areas dissected 24 h later were measured on a gamma counter (Figure 1C,D). CDNF diffused most strongly to the ipsilateral subthalamic nucleus (STN) from the SN. The radioactivity in the ipsilateral SN was the highest overall, with it being significantly different than the other brain areas (excluding the STN) [One-way ANOVA, *F*(11,52) = 8.6, *p* = < 0.0001] (Figure 1C).

To study whether the spread of CDNF to the STN was due to passive diffusion or active transport mechanisms, ^125^I-CDNF was administered to the SN together with two doses of unlabelled CDNF (2000 fold molar excess or 10 000 fold molar excess) to compete for binding and active axonal transport of the labeled CDNF. The diffusion/transport of ^125^I-CDNF from the SN to the STN was not blocked by either of the doses of unlabelled CDNF [One-way ANOVA, *F*(2,18) = 0.0068, low: *p* = 0.9801, *df* = 52; high: *p* = 0.9928, *df* = 52] (Figure 1E) which suggests passive diffusion of CDNF after nigral administration.

### Diffusion of Unlabelled CDNF Protein in the Naïve Rat Brain After Nigral Administration

**FIGURE 2:**
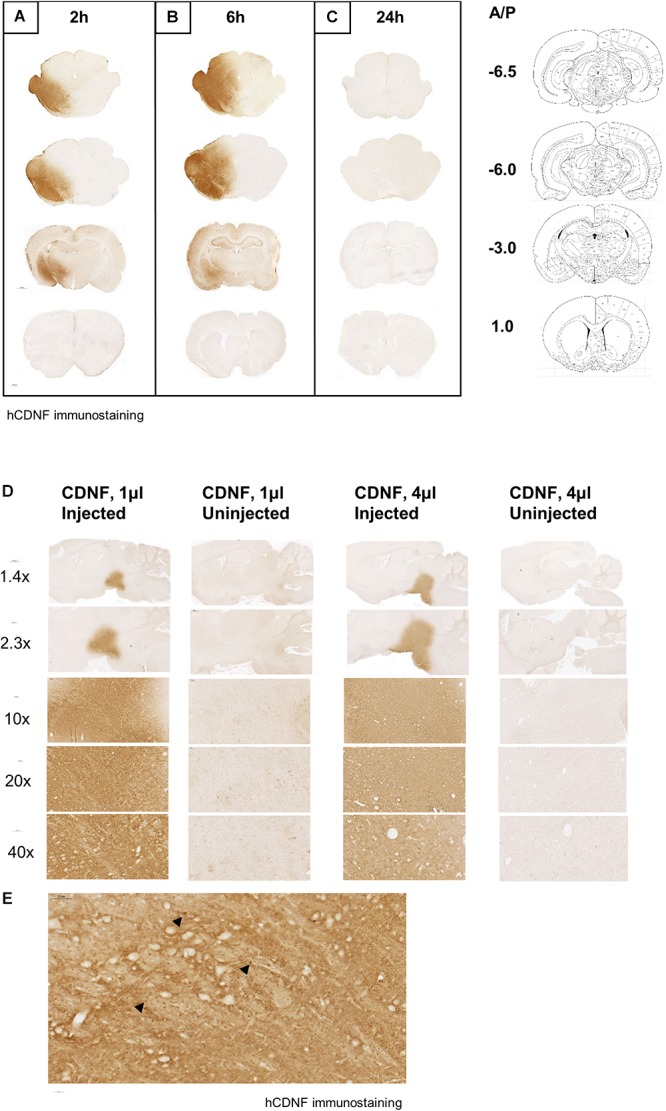
Diffusion of human CDNF after injection to the SN in naive rat brain at different time points and volumes. CDNF (3 μg in 4 μl) was injected to the SN of rats and perfused **(A)** 2, **(B)** 6, or **(C)** 24 h later. Human CDNF (hCDNF) immunostaining of a representative posterior, nigral, thalamic, and striatal section for each time point. *N* = 2/time point. Diffusion of human CDNF (3 μg) when injected into the SN in the volume of 1 or 4 μl. Rats were perfused 2 h after the injection. **(D)** Increasing magnification of CDNF injected side versus the uninjected side of the injected area. **(E)** 40× maginification of injected area. Black arrowheads indicate dark brown puncta staining of CDNF.

When CDNF protein was injected to the SN of the naïve rat brain and monitored at different time points in order to characterize the diffusion after nigral injection, we observed robust CDNF staining in the ipsilateral midbrain including the SN at the 2 (Figure 2A) and 6 (Figure 2B) hour time points, but not at the 24 h time point (Figure 2C). Additionally, CDNF protein appeared to diffuse anteriorly and posteriorly from the SN at 2 and 6 h, but again was not detected at 24 h. At the 2 and 6 h time points, there was clear staining in the hippocampus and amygdala, as well as thalamic and hypothalamic regions. There was no staining observed in the dorsal STR at any of the time points. As this was a qualitative experiment, two rats were used per time point and the staining was similar in both.

Additionally, increasing the volume of the CDNF injection while keeping the total amount of protein constant, increased its diffusion in the SN. When 1 or 4 μl of CDNF was injected to the SN of naïve rats, the higher volume appeared to diffuse farther after 2 h as compared to the lower volume (Figure 2D). It can also be observed that at higher magnifications there are concentrated, darkly stained puncta throughout the injected area (Figure 2E, black arrowheads).

### CDNF Colocalizes With TH+ Neurons and Is Diffused Around the Nigral Area

**FIGURE 3:**
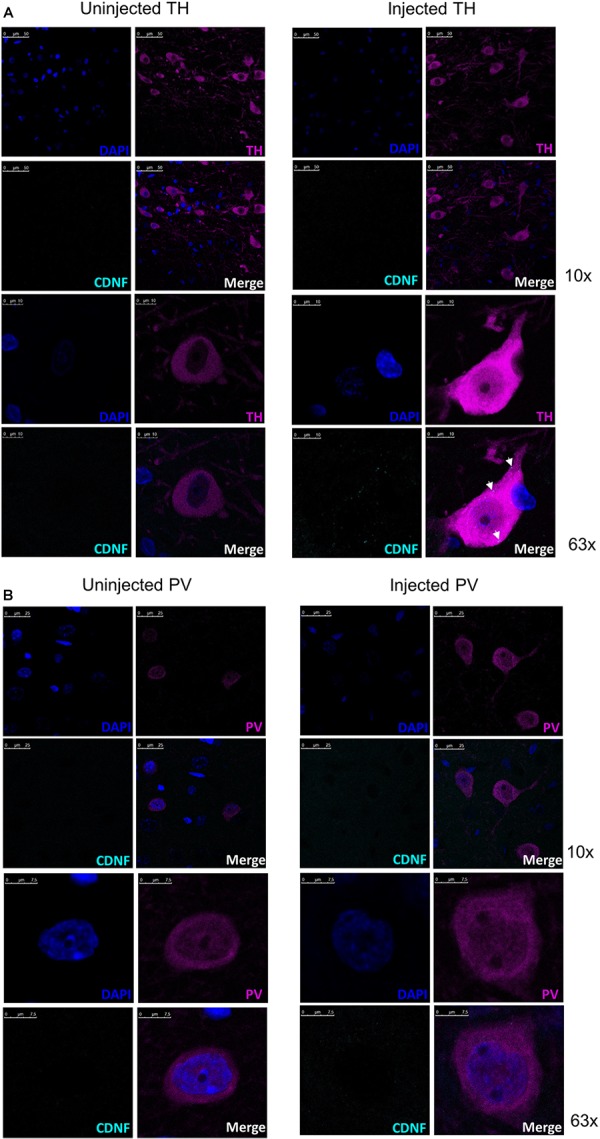
Representative confocal images of CDNF-injected rat substantia nigra with tyrosine hydroxylase (TH) neurons or parvalbumin (PV) neurons to demonstrate colocalization. **(A)** Representative confocal images from a rat brain injected with human CDNF into the substantia nigra (SN) and perfused 2 h later with costaining for CDNF and TH. Total of six rats were injected and stained for the colocalization study. White arrows in the injected side indicate CDNF immunoreactive puncta (cyan dots). Uninjected side representative image taken with identical settings to account for possible background. Top panel 10× objective, lower panel 63× objective. **(B)** Representative confocal images from rats injected with human CDNF into the SN and perfused 2 h later with costaining for CDNF and PV. Uninjected side representive image taken with identical settings to account for possible background. Top panels 10× objective, lower panels 63× objective. *N* = 6.

As shown above (Figure 2A), 2 h after the injection to the SN CDNF staining is present in the nigral area. Next, we wanted to determine whether CDNF colocalized with TH+ neurons or parvalbumin (PV) neurons using immunofluorescent staining and confocal microscopy. Brains from six rats were analyzed to clarify whether CDNF colocalized with either of the neuronal subtypes. Out of several cells detected in the nigral area of each brain, it could be observed that there were CDNF immunoreactive puncta (cyan, white arrows) inside approximately two thirds of the TH+ neurons (magenta, Figure 3A) on the injected side. In contrast, out of several cells observed in the PV stained sections, CDNF was diffused around the neurons but did not appear to colocalize with them (Figure 3B), since only approximately one tenth of the PV+ cells had immunoreactive puncta present. Additionally, as can be clearly observed in Figure 2, there is diffuse CDNF staining all around the midbrain/SN. The confocal images show the same: CDNF is diffused around the cells and only some cells have CDNF-immunoreactive puncta.

## Discussion

We showed that radiolabelled CDNF injected into the SN was not transported to the STR or other brain areas, whereas striatally injected CDNF was transported to SN, hippocampus, and frontal cortical areas as shown earlier by Voutilainen et al. (2011). Instead, CDNF readily diffused in the brainstem/midbrain, in particular around the SN, to the hippocampus, and amygdala within 2 to 6 h after the nigral injection. Additionally, CDNF colocalized with TH+ neurons in the SN, indicating it was likely taken up by these neurons.

Since diffusion and transport of CDNF after striatal injection has been studied previously, we wanted to investigate the diffusion of CDNF after nigral injection. Using radiolabelled CDNF we observed the spread of the protein to distinct brain regions. As shown earlier by Voutilainen et al. (2011), when injected to the STR, CDNF spread to the cortex, both sides of the STR, and the hippocampus. There is also clear evidence of CDNF transported to the SN after striatal injection (Voutilainen et al., 2011; Matlik et al., 2017). After injection of radiolabelled CDNF to the SN, it remained in the ipsilateral side and only diffused as far as the STN. CDNF did not seem to be transported or diffused to the STR. This is in line with the immunohistochemistry data obtained here. When unlabelled protein was used to block the transport of radiolabelled CDNF after nigral injection, neither the 2000 nor the 10 000 molar excess of unlabelled protein could block the diffusion of CDNF to the STN. Therefore, we can conclude that CDNF is not undergoing active transport, but rather passive diffusion after nigral administration. Interestingly, only radiolabelled CDNF was present in the brain regions 24 h after the injection. The discrepancy between the radioactivity measurements and the immunohistochemistry results could be due to the limit of detection for the antibody used. In other words, though the CDNF antibody is able to detect the injected human CDNF specifically, it may be that it has diffused significantly or degraded by this time point, and was not visible by eye or microscope in the immunostained brains.

Since radiolabelled CDNF is detected significantly in the STN of the brains, it seems that it is diffusing to this area, and is not transported, as indicated from the blocking study. Nonetheless, there is the possibility that CDNF was taken up by glutamate terminals of the SN reticulate and transported to the STN (Robledo and Feger, 1990; Parent and Hazrati, 1995). Although this was not studied here, future studies could examine the potential uptake and effects of CDNF at the STN, which might be critical for the biological effects of extracellularly administered CDNF.

The same phenomenon observed in the current study is also occurring when GDNF, a neurotrophic factor with known transmembrane receptor GFRalpha1/RET, is administered to the STR or SN. After striatal delivery, GDNF is transported to the SN whereas with nigral delivery it diffuses in the SN area as well as the hippocampus but is not transported to the STR (Kearns and Gash, 1995; Tomac et al., 1995). This then begs the question why are these proteins being transported to the SN from STR but not vice versa when it has been demonstrated that in other contexts molecules are transported from SN to STR (Hattori et al., 1973). Instead, CDNF may undergo lysosomal degradation when injected near the cell bodies, instead of axonal transport as is the case with nerve growth factor (Butowt and von Bartheld, 2009).

When recombinant human CDNF was injected to the SN of naïve rats, it was detected widely in the midbrain areas after 2 and 6 h, but not at 24 h post-injection. This could indicate that the protein was cleared away between 7 and 24 h, or that it is diffusing significantly and therefore is at a lower concentration which could not be detected anymore using immunostaining. Since the half-life of CDNF is 5.5 h after striatal injection (Matlik et al., 2017), this is expected. In the representative sagittal images of the CDNF staining in paraffin sections, CDNF appears to diffuse farther with a larger volume though the protein amount is the same. These results indicate the importance of volume in CDNF’s diffusion, where a higher volume may lead to the protein itself reaching a more distant area to exert its effects, even if the concentration is lower. This has also been observed with GDNF (Taylor et al., 2013).

Lastly, when the midbrain sections were co-stained for CDNF and TH/PV, we observed that CDNF did colocalize with TH positive neurons in the SN, while the colocalization with PV positive neurons was less evident. Colocalization with TH-expressing cells in the SN was also observed by Matlik et al. (2017). They showed CDNF to be present in the TH+ neurons 6 h after striatal injection. PV neurons present in the SN (Gerfen et al., 1985), of which most are GABAergic inhibitory neurons (Lee and Tepper, 2007), were chosen as another neuron subtype for colocalization due to their putative involvement in sensorimotor functions and motor integration (Bernacer et al., 2012), which are important for PD. Based on our results, it appears that CDNF is not colocalizing with PV+ neurons in the SN. Thus, CDNF may exert its effects at the cell surface since it is present around the cells. Since the colocalization of CDNF with different neuronal subtypes was assessed only qualitatively here, further analyses are needed to draw decisive conclusions.

The diffusion of CDNF after injection to the SN was studied here for the first time. We observed clear diffusion of CDNF to the STN and the brainstem areas around the SN, but no transport to the dorsal STR. Interestingly, we observed colocalization of CDNF with TH+ neurons, but less so with PV+ neurons, in the SN. Knowing the optimal site of administration and diffusion or transportation properties of a neurotrophic treatment is of paramount importance in developing new disease-modifying therapies for PD where the normal neuroanatomical connections are lost due to degenerating dopamine neurons.

## Ethics Statement

All animal experiments were approved by the Finnish National Board of Animal Experiments (ESAVI/5459/04.10.03/2011 and ESAVI/7812/04.10.07/2015) and were carried out according to the European Community guidelines for the use of experimental animals.

## Author Contributions

KA participated in the planning and execution of all the experiments, and wrote the manuscript. J-MR participated in the experiments, and commented on the manuscript. KM helped to plan and gave advice on the colocalization experiments. MA guided the experiments, helped with manuscript preparation, and participated in the experiments. MV participated in the planning and execution of all the experiments, and helped to prepare the manuscript.

## Conflict of Interest Statement

MV is an inventor in a CDNF-related patent application that is owned by Herantis Pharma Plc. The remaining authors declare that the research was conducted in the absence of any commercial or financial relationships that could be construed as a potential conflict of interest.
